# 

**Published:** 1893-08

**Authors:** 


### CONTENTS—AUGUST, 1893.—VOL. IX., NO. 2.

ORIGINAL COMMUNICATIONS—
A Case of Railway Spine. Allen J. Smith, M. D., Galveston . . . 51
Report of a Case of the Difficulties met with in Primiparous Women. A. E. Garrett, M. D., Sulphur Springs . . . . . . . 57
Spurious Pregnancy. Bruce P. McVey, M. D., Mumford . . . . . 60
Atypical Forms of Disease as Influenced by Environments. L. M. Stroud, M. D., Terrell . . . . . . . 62
Removal of an Ovarian Tumor, weighing 111 pounds, from a Child. W. W. Keen, M. D., Philadelphia . . . . . . . 64
The Outlook of the Profession to the Young M. D. Peel M. Payne, M. D., Terrell . . . . . . . 67
Ulcer of the Foot Healed by Specifics and Thiersch's Method of Skin Grafting. E. B. Jackson, M. D, Houston . . . . . . . 68

ABSTRACTS OF CURRENT MEDICAL LITERATURE—
Department of Practice of Medicine. Prof. A. J. Smith, Galveston . 68
Department of Therapeutics. Dr. David Cerna, Galveston . . . . . 72
Department of Dermatology. Dr. Isadore Dyer, New Orleans . . . 75

CORRESPONDENCE—
Secretary West and the Buffet Banquet. D. R. Wallace, M. D., Waco 78

SOCIETY NOTES—
Pan-American Medical Congress . . . . . . . 80

EDITORIAL—
Intelligent Treatment of Domestic Animals . . . . . . . 81
Function of the Pharmacist . . . . . . . 83
Texas Delegates to Pan-American Medical Congress . . . . . . . 86

MEDICAL NEWS AND MISCELLANY—
Numerous paragraphs of general interest to the profession . . . . . . 87

BOOK NOTICES—
Diseases of the Nervous System . . . . . . . 80
A Text-Book of the Theory on Practice of Medicine . . . . . . . 92
Lessons in Practical Diagnosis . . . . . . . 93
PUBLISHERS' NOTES . . . . . . . 46

